# Monitoring mood state to improve performance in soccer players: A brief review

**DOI:** 10.3389/fpsyg.2023.1095238

**Published:** 2023-02-22

**Authors:** Okba Selmi, Ibrahim Ouergui, Antonella Muscella, Danielle E. Levitt, Katsuhiko Suzuki, Anissa Bouassida

**Affiliations:** ^1^High Institute of Sports and Physical Education of Kef, University of Jendouba, El Kef, Tunisia; ^2^Research Unit: Sportive Sciences, Health and Movement, El Kef, Tunisia; ^3^High Institute of Sports and Physical Education, Ksar Said, University of Manouba, Tunis, Tunisia; ^4^Department of Biological and Environmental Science and Technologies, University of Salento, Lecce, Italy; ^5^Department of Kinesiology and Sport Management, Texas Tech University, Lubbock, TX, United States; ^6^Faculty of Sport Sciences, Waseda University, Saitama, Japan

**Keywords:** soccer, affect, exercise training, psychology, athletic performance

## Abstract

**Introduction:**

Psychological aspects of sport are key in maintaining athlete motivation and make a difference in competitive outcomes. Adjustments to training may be necessary according to athletes’ emotional state. Therefore, it is important to assess and quantify mood states throughout the season in team sports, including among soccer players. The Profile of Mood States (POMS) is a widely used questionnaire that assesses emotional states characterized by positive or negative feelings and can be administered repeatedly to assess changes in mood state. This review aims to assess and summarize the current literature on mood state variation in soccer players with a specific focus on training loads, training modalities, and competitive performance.

**Methods:**

A literature search was systematically conducted and resulted in 156 records. After removing duplicates, items with irrelevant titles and abstracts were screened out, and full texts were then screened for relevance and compared with inclusion and exclusion criteria. The remaining 37 articles were included in the final qualitative synthesis.

**Results:**

POMS scores were related to variability in training load, intensity of the training period, modality of training exercises, competitive performance and time of day in soccer players. Common recommendations include monitoring the mood state of soccer players during training sessions, matches, and throughout training periods to detect early signs of psychological disturbance and aid in optimizing high-level training performance.

**Conclusion:**

The POMS allows for monitoring of players’ psychological state, providing coaches with data to aid in adjusting acute program variables according to players’ psychological states and improve performance. Results offer practical support for the use of a simple POMS measurement as part of an overall program to monitor the players’ psychological states. Results also highlight how training choices (i.e., load and exercise modality) and competitive performance are related to mood states (i.e., tension, anger, confusion, depression, fatigue, and vigor).

## 1. Introduction

Optimization of sport performance is linked to multiple factors such as physical, technical, tactical, and cognitive skills. The importance of mental health as a factor in sport performance is becoming increasingly recognized, including among soccer players (Soylu et al., 2021). Mental health is a concept that includes emotional, psychological and social states of athletes and affects the way they feel, react, and behave (Souter et al., 2018; Russell et al., 2020). Among the many dimensions of mental health is mood state which may change in response to the daily and periodic challenges that soccer players face (Selmi et al., 2017). Additive stressors from soccer players’ personal lives, wellbeing, and sport training can influence mood state (Yagmaee, 2021). It is clear that soccer performance is closely linked to mental health in general and to mood state specifically (Yagmaee, 2021).

It is well known that in the field of sport, mood state is affected by psychophysiological responses (Soylu et al., 2021) and influenced by various factors such as training modality (Selmi et al., 2017), training load (TL; Beykzade et al., 2011; Watson et al., 2017; Botelho et al., 2022), competitive performance (Filaire et al., 2001; Casanova et al., 2016) and motivation and concentration of athletes during training (Aydi et al., 2022). Therefore, sport psychologists have sought to develop and validate reliable tools and inventories to assess athletes’ mood states (Filaire et al., 2001; Saidi et al., 2020). In fact, the validated Profile of Mood States (POMS) questionnaire has been widely used to assess mood state in different sport situations such as during individual training sessions, throughout training periods, and before or after competitions (Miranda et al., 2013; Saidi et al., 2020; Selmi et al., 2020). Additionally, this tool has been widely used to explore the relationship between training modalities, TL and athletes’ psychological state (Lovell et al., 2010; Aydi et al., 2022). For that reason, it is suggested that psychological state should be assessed during training to control and prevent negative mood states that are related to training intensity and exercises’ modalities.

Increased TL during intense training periods in soccer is associated with a lack of concentration, negative feelings, uncertainty, and mental fatigue (Smith et al., 2016), which are reflected by mood and behavior changes, increased anxiety and aggressiveness, indifference, irritability, and sleep disturbance (Selmi et al., 2020). Sleep quality is a key and vital factor which can affect athletes’ mood state (Paryab et al., 2021). In fact, sleep loss is detrimental to vigor and attention and increases fatigue, confusion, depression, and tension (Benjamin et al., 2019, 2020). Moreover, sleep is essential to maintain adequate recovery and stress tolerance among soccer athletes, and needs increase with TL (De Morais Ferreira et al., 2022). Because of the potential implications of psychological aspects of sport, a great deal of attention has been focused on the impact of high TL on mood. Several studies have confirmed that mood disturbances increase during heavy training periods (Smith et al., 2016; Botelho et al., 2022) alongside physiological variations (Filaire et al., 2001; Silva et al., 2008). Based on this body of work, Smith et al. (2016) suggested that negative affective responses and reduced performance may be related to physical fatigue accumulation, while positive affective responses during the taper period were associated with positive physiological changes and higher performance (Filaire et al., 2001; Beykzade et al., 2011). This is likely due to fatigue reduction and recovery during taper which allow for improved physical performance (Schmikli et al., 2011).

Additionally, mood state variation has been associated with performance achievement or failure (Jones et al., 2010; Miranda et al., 2013; Casanova et al., 2016; Picoli and Bueno, 2022). Therefore, mood regulation strategies may be beneficial for the competitive performance among soccer players (Hashim et al., 2011; Bijukumar, 2021). Mood regulation is associated with training modalities and player motivation (Selmi et al., 2017). In fact, verbal encouragement, a form of motivational training, improved mood state and resulted in greater self-reported physical enjoyment among young soccer players (Aydi et al., 2022). Using the POMS to monitor players’ mood state may allow the technical staff of the soccer teams to better adapt TL, improve programming and select motivational training exercises to maximize performance *via* improved mood state.

This brief review summarizes the current literature on mood state variation in soccer players in the following areas: (1) TL and training period; (2) exercises modality; (3) competitive performance; and time of day. Additionally, a discussion of the impact of training on mood state is presented. Practical applications and future research are suggested.

## 2. Materials and methods

### 2.1. Search strategy

**Figure 1:**
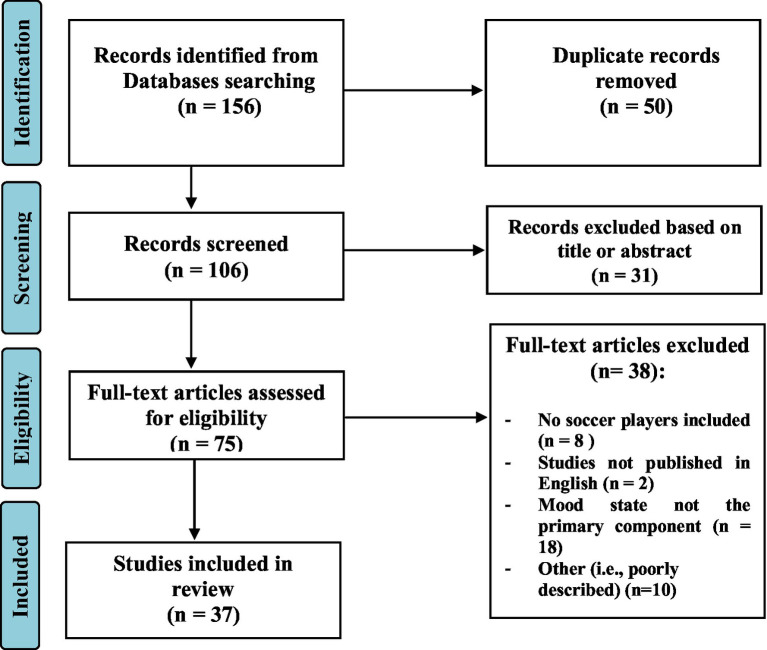
Flowchart describing the study selection process.

This review included studies that examined the effect of training on mood state within soccer players. The literature search was performed using PubMed, Scopus, Web of Science, and Google Scholar and included articles published until August 10, 2022. The oldest article retrieved was published in 2001. Moreover, we performed manual searches of relevant journals and reference lists obtained from published articles. The following English terms and key words were searched using Boolean operators: “mood state” AND “soccer” combined with the key words “training load,” “training period,” “training exercise” and “performance”(i.e., “mood state” AND “soccer” AND “training load,” “mood state” AND “soccer” AND “training period,” “mood state” AND “soccer” AND “training exercise,” and “mood state” AND “soccer” AND “performance”). The inclusion criteria for these articles were: (1) the original studies were published in English; (2) data concerning training load, training period, exercise training or performance; (3) players included soccer players; and (4) studies examined mood state. The exclusion criteria were: (1) studies that included sports other than soccer; (2) studies that did not include any of the parameters evaluated in this review; and (3) reviews, commentaries, interviews or expert opinions, posters, or book chapters (Figure 1).

Two authors independently extracted and reviewed study data to verify if a given study met the inclusion criteria. Disagreements between the two authors on the inclusion criteria were resolved by a third author with experience in the field.

### 2.2. Definition and measurement of mood state

According to Lane (2001), mood is a set of sensations varying in intensity and duration. Chennaoui et al. (2016) reported that emotional mood contributes to an athletes’ likelihood of success or failure in relation to training. This concept refers to the change in emotional state. Athletes may think of this dichotomously and report being in a good or bad mood characterized by overall positive or negative feelings, respectively (Selmi et al., 2020).

To identify practical assessments, researchers examined various psychological dimensions related to training that require less technology such as mood state questionnaires which have been actively researched in recent years (Saidi et al., 2020; Aydi et al., 2022; Picoli and Bueno, 2022). The profile of mood states (POMS) is a self-report questionnaire comprised of 65 items assessing seven mood states subscales (i.e., tension-anxiety, anger-hostility, confusion-bewilderment, depression-dejection, fatigue-inertia, vigor-activity, and interpersonal relationship; Table 1), with the interpersonal relationship subscale often being eliminated from measurement by researchers. Each item is rated on a 5-point Likert scale from 0 (not at all) to 4 (extremely) in response to questions such as “How are you feeling right now? (Aroian et al., 2007). The six subscales of POMS can be combined into a total mood disturbance (TMD) score by summing the (T) scores for the five negative mood subscales and subtracting the T score for positive mood state and adding a constant of 100 in order to prevent negative numbers.

**Table 1:** Summary of items making up each subscale.

Subscales	Item numbers	Total # of items	Range of scores
Tension-anxiety (TEN)	2, 10, 16, 20, 22*, 26, 27, 34, 41	9	0–36
Anger-hostility (ANG)	3, 12, 17, 24, 31, 33, 39, 42, 47, 52, 53, 57	12	0–48
Confusion-bewilderment (CONF)	8, 28, 37, 50, 54*, 59, 64	7	0–28
Fatigue-inertia (FAT)	4, 11, 29, 40, 46, 49, 65	7	0–60
Depression-dejection (DEP)	5, 9, 14, 18, 21, 23, 32, 35, 36, 44, 45, 48, 58, 61, 62	15	0–28
Vigor-activity (VIG)	7, 15, 19, 38, 51, 56, 60, 63	8	0–32
Interpersonal relationship (REL)	1,6, 13, 25, 30, 43, 55	7	0–28
Total Mood Disturbance (TMD)	(TEN + ANG + CONF + FAT + DEP) – VIG + 100	-	68–300

Terry et al. (2003) published guidelines for the definition of each emotional state assessed by the POMS. According to this work, tension is characterized by sensations such as nervousness, apprehension, worry and anxiety. Anger is characterized by feelings that vary in intensity from annoyance or mild aggravation, fury, and irritation. Confusion described feelings of indecision associated with a decreased ability to control alertness and emotions. Depression is associated with a negative self-concept characterized by themes such as disappointment, deficiency, ineffectiveness, and self-blame. Fatigue is characterized by perception of mental and physical fatigue. Vigor represents the state of well-being, power, excitement, concentration, alertness, vitality, and strong ability to execute physical and mental effort. Finally, interpersonal relationships describe more of a trait than a specific state, but a strong score on this subscale is considered to indicate positive emotional state (Terry et al., 2003). These dimensions can be expressed in terms of state or trait depending on whether the instructions accompanying the form guide the participant to concentrate on their feelings at the moment, throughout the day or week of the assessment, or their usual feelings.

Some researchers have distinguished between high performers and low performers based on their pre-competition mood states (Filaire et al., 2001; Casanova et al., 2016). These studies reported that positive emotional state and successful athletic performance are strongly correlated. Specifically, the players who present lower tension, depression, anger, confusion and fatigue scores and higher vigor scores generally have more competitive success than players with the opposite profile as assessed by the POMS questionnaire. This positive profile has been called the “iceberg” profile (i.e., high vigor score and low scores for depression, tension, fatigue, confusion and anger) (Filaire et al., 2001; Casanova et al., 2016).

## 3. Results

### 3.1. The relationship between training loads, training periods, and mood state variation

During high-intensity training periods characterized by heavy TLs, athletes have usually reported significant mood changes (Filaire et al., 2001; Lovell et al., 2010; Picoli and Bueno, 2022). For example, Filaire et al. (2001) showed that mood disturbance increased progressively with higher TL. It has also been shown that acute fatigue during intense training periods was associated with worse mood state and decreased vigor (Lovell et al., 2010; Saidi et al., 2020). Similarly, Fields et al. (2021) reported higher intensities during soccer preseason were associated with more negative mood scores (i.e., anxiety, anger, depression, confusion, fatigue), while indicators of positive mood states (i.e., vigor) decreased. These results are in line with those reported by Filaire et al. (2001), which showed that mood disturbance increased with the training stimulus. Amstrup et al. (2002) examined the effects of sports season on mood states and found no change in POMS scores during the pre-season period. However, vigor decreased and fatigue and depression increased from the end of pre-season to mid-season. Additionally, Miranda et al. (2013) studied changes in mood across of10 weeks of training in young soccer players. This study showed that as training intensity increased throughout the training period, the mood state became more negative (Miranda et al., 2013). However, Silva et al. (2008) reported that POMS scores were stable after12 weeks of high training intensity, suggesting that athletes’ mood states may be more vulnerable during extended periods of intense training.

The relationship between overtraining and mood state has also been investigated. Schmikli et al. (2011) reported a significant increase in depression and anger subscale scores and total mood disturbance (TMD) with overreaching in soccer players. Lovell et al. (2010) found that mood state shifted from more positive to more negative as the competitive season progressed, likely due to increasing TL and accumulating stress. Similarly, during a period of congested matches (10 games over 6 weeks) soccer players reportedly significantly increased fatigue, tension, anger and a decreased vigor compared to the non-congested period of match play (6 games over 6 weeks). They suggested that mood state disturbance is associated with increased physical and physiological load. Additionally, Botelho et al. (2022) showed significantly increased negative mood scores (i.e., tension, depression, anger, fatigue, confusion, and TMD) after7 weeks of intensive pre-season training in female soccer players, suggesting that training intensity increase may be associated with negative mood changes (Filaire et al., 2001). Results from these studies are consistent with the finding that substantial increases in training intensity may result in a decreased ability to concentrate and feelings of disorder, uncertainty, and mental fatigue (Djaoui et al., 2017). These changes in mood are also associated with poorer recovery state and well-being changes in soccer players (Selmi et al., 2020).

The tapering period, characterized by reduced training intensity, generally appears to have a positive effect on mood state. Beykzade et al. (2011) reported that after the tapering period, TMD, fatigue and depression decreased significantly compared to the beginning of the intense training period. Reducing the training intensity during the tapering period can produce a balance between effort and recovery which leads to mood regulation (Beykzade et al., 2011).

Regarding the study during Ramadan, the study by Baklouti et al. (2017) compared the effects of two small-sided game (SSG) training formats (4 × 4 min and 2 × 8 min) on mood state during Ramadan in male professional soccer players. The results indicated that Ramadan and not training increased subjective feelings of fatigue. Along the same lines, the study by Chtourou et al. (2011) which examined the effects of Ramadan fasting on RPE and mood state. They indicated that RPE and fatigue score were higher during Ramadan in comparison with before Ramadan.

Overall, results from studies using the POMS to assess mood state reveal that extended intense training is associated with more negative mood states, whereas periods of reduced training can be useful in restoring more positive mood state. However, short-term intense training may not negatively impact mood state. A summary of 13 studies examining the effects of both training intensity and periods on mood state variation is presented in Table 2.

**Table 2:** Findings from studies examining mood variations with different training periods or intensities.

Study	Participant (number, sex, level, age)	Condition/ duration	Aim	Results	Findings
Amstrup et al. (2002)	20, male, professional (20.0 ± 1.2 years)	Sports season	To monitor mood states (POMS) at the start and end of pre-season training, mid-season and at the end of competitive season	No change in POMS scores during the pre-season period decrease in vigor and increases in fatigue and depression from end of pre-season to mid-season	Mood disturbance at the mid-season with decline in vigor and increases in fatigue and depression
Baklouti et al. (2017)	24, male, professional (24 ± 4 years)	Ramadan	To compare the effects of two small-sided game (SSG) training formats (4 × 4 min (SSG-S) and 2 × 8 min (SSG-L)) on mood state and RPE	Compared to before Ramadan, fatigue score was higher at end of Ramadan. RPE measured during Ramadan were higher after SSG-L than SSG-S sessions	Ramadan and not training increased subjective feelings of fatigue
Beykzade et al. (2011)	15, male, amateur (25.0 ± 2.2 years)	Pre-season (5 weeks)	To assess the mood profile during pre-season preparatory	No significant change in mood after 4 weeks of progressive training load, except for fatigue score. After the taper period (1 week), fatigue and depression scores showed significant decrease compared to the beginning of the training period	Fatigue and depression have been shown to be more sensitive to the change of training load
Botelho et al. (2022)	24, female, elite (26.0 ± 3.7 years)	Preseason (7 weeks)	To investigate changes and correlations between mood states and various physiological stress markers	POMS scores (except vigor), testosterone, and cortisol concentrations, as well as CK, showed significant Changes after the preseason. Correlations were found between cortisol and tension, cortisol and confusion	Coaches and physical coaches can use these data to monitor, and control Training load and training programs, in particular throughout the preseason period
Chtourou et al. (2011)	20, male, young soccer players (17.6 ± 0.6 years)	Ramadan	To assess the effects of Ramadan fasting on RPE and mood state	RPE and fatigue score were higher during Ramadan in comparison with before Ramadan	RPE and feelings of fatigue were higher during Ramadan
Fields et al. (2021)	20, male, collegiate (20.3 ± 0.9 years)	Pre-season	To examine the relationship between external load and mood state during soccer preseason	Morning ratings of negative mood were positively predicted by previous day’s afternoon practice high-speed distance. In addition, negative morning mood states inversely predicted high-speed distance, Total distance, and player load for that day’s afternoon practice	Using POMS questionnaire with GPS may improve the understanding of physical and psychological responses
Lovell et al. (2010)	16, male, professional (25.0 ± 5.0 years)	Competitive season	To assess whether the demands of the modern English competitive soccer season would be reflected in the mood states	At the start of the season, the players had a positive mood state, however, as the season progressed, they showed a shift toward a negative mood state	The extended competitive season, increased training load and increase in stress lead to negative mood
Miranda et al. (2013)	13, male, youth players (17.0 ± 0.7 years)	At the beginning of the season (10 weeks)	To examine the influence of 10-week soccer training program on mood state	The positive score (i.e., vigor) was reduced, the negative scores (i.e., tension, depression, anger, fatigue, confusion and TMD) were increased	Mood disturbance is associated with long period of high training load (volume and intensity)
Silva et al. (2008)	15, male, professional (23.4 ± 2.5 years)	Competitive period (12 weeks)	To investigate the change of mood state in Brazilian soccer players during a training program	Decrease in vigor score in T3 (week 12) compared with T1 (week 0) and T2 (week 6)	The training program intensified between T2 and T3 led to the reduction of the positive mood score (vigor)
Saidi et al. (2020)	24, male, elite (19 to 22 years)	6 weeks after the beginning of the competitive period (12 weeks)	To analyze mood state in relation to changes in training and match exposure during a congested period of match play	A significant increase was found in fatigue, tension, anger and a significant decrease in vigor from the non-congested period of match play when the players played 6 games over 6 weeks to the congested period when the players played 10 games over 6 weeks	Mood state monitoring could be a practical and efficacy tool to verify the degree of preparedness for match play during a congested period
Schmikli et al. (2011)	77, male, young elite (16–21 years)	Competitive season	To examine the effects of performance decrement on mood during overreaching	The performance decrement group scored higher on depression and anger than controls	Disturbance in mood state is associated with the performance decrement of players during overreaching
Selmi et al. (2020)	24, male, professional (17.0 ± 0.2 years)	Pre-season (7 weeks)	To compare the effects of two intensified training period on mood state	Negative POMS scores increased (tension, anger, confusion, depression, fatigue and vigor decreased)	intensified training period affect negatively mood state

### 3.2. Training exercises and mood state

Limited research has investigated how acute mood state changes with different training exercises in soccer. Those that have indicate that intense training exercises can change mood state in soccer players (Selmi et al., 2017). In fact, Selmi et al. (2018) assessed mood responses to high-intensity interval training (HIIT) in soccer players, with the POMS questionnaire administered immediately before and within 5 min after training. Results indicated a significant increase in negative mood scores (i.e., anxiety, fatigue, and global mood score) and decrease in vigor score. Selmi et al. (2017) also reported that physical small-sided games (SSGs) induced significant improvements in mood state in professional soccer players. In the same study, both HIIT and SSG sessions induced similar physiological responses; in contrast, HIIT resulted in mood disturbance highlighted by increased tension, fatigue and TMD scores, whereas SSGs provided mood balance. The stability of POMS scores in this study may be due to athletes’ motivation during preferred exercise modalities (i.e., exercises using the ball; Selmi et al., 2017). Similarly, Oliveira et al. (2009) reported that fatigue scores increased following a HIIT session in female soccer players. Broodryk et al. (2017) examined the effect of an anaerobic fatigue test (Yo-Yo Intermittent Recovery) on cortisol levels and mood state in female semi-professional soccer players and found significantly increased cortisol, psychological fatigue and TMD and decreased vigor. Sparkes et al. (2018) found perturbed mood state for up to 24 h after intensive SSG during the competitive period in professional soccer. Exercise motivation is associated with positive mood in soccer players and may be achieved using specific drills such as integrating the ball during physical tasks or the use of verbal encouragement by coaches (Aydi et al., 2022). Specifically, Aydi et al. (2022) showed that technical circuit dribbling drills with verbal coach encouragement improved mood state highlighted by decreased scores on negative mood subscales and increased vigor. Together, these studies suggest that verbal encouragement is an effective method to improve mood or prevent mood disturbances during training.

A summary of 5 studies investigating the relationships between exercise session characteristics and mood state variation is presented in Table 3.

**Table 3:** Findings from studies examining relationships between training exercises and mood state.

Study	Participant (number, sex, level, age)	Condition/ duration	Aim	Results	Findings
Aydi et al. (2022)	16, male, soccer specialist students (17.7 ± 0.5 years)	Mid-season (2 weeks)	To investigate the effects of verbal encouragement (VE) on mood state and physical enjoyment during a soccer dribbling circuit exercise (the Hoff circuit)	The circuit exercise without VE showed lower vigor and higher total mood disturbance and was associated with higher tension and fatigue, compared to the circuit exercise with VE	The soccer dribbling circuit exercise with VE condition resulted in positive mood state, compared to that of the soccer dribbling circuit exercise without VE
Broodryk et al. (2017)	47, female, semi-professional (22.0 ± 2.7 years)	Competitive training period	To examine the effects of anaerobic fatiguing test (5-m multiple shuttles run test) in mood state	Increase in fatigue and TMD from pre to post test. Decreased in vigor and confusion from pre to post test	The anaerobic fatiguing test can be perceived as a psychological stressor by female players
Selmi et al. (2017)	16, male, professional, (24.1 ± 0.9 years)	Competitive period	To compare the effects of high-intensity intermittent training (HIIT) versus small-sided games (SSG) on the mood state	The HIIT compared with SSG resulted in: an increased total mood disturbance fatigue, tension decreased positive mood (vigor)	SSG ensured mood balance while HIIT produced a mood disturbance
Selmi et al. (2018)	20, male, amateur (23.9 ± 0.9 years)	Competitive period	To examine the effects of HIIT on mood state and to show the relationship between RPE and POMS scores	HIIT leads to an increase in anxiety, fatigue, total mood disturbance and a decreased vigor. No correlation was found between POMS scores and RPE	HIIT causes negative mood changes that trainers should consider optimizing fitness and recovery
Sparkes et al. (2018)	16, male, professional (21 ± 2 years)	Competitive season	To characterize the mood response to a SSG exercises over 24 h	Immediate disturbances in mood following 42 min of SSG and + 2 h. On the following morning (+24 h), POMS scores had returned to baseline values	SSG exercises result in a perturbation in mood state for up to 24 h

TMD: total mood disturbance; SSG: small-sided games; HIIT: high-intensity interval training; RPE: ratings of perceived exertion.

### 3.3. Competitive performance and mood state

Previous studies have examined the relationship between mood state and performance using the POMS questionnaire (Filaire et al., 2001; Miranda et al., 2013). In fact, mood state in soccer is related to competitive performance in terms of win/draw/lose (Casanova et al., 2016). Studies with professional soccer players have shown that athletic performance can improve positive and decrease negative POMS subscale scores (Robinson and Howe, 1987; Filaire et al., 2001; Miranda et al., 2013). Furthermore, Silva et al. (2008) showed that when professional team presented an “iceberg” profile during the training program, with a fatigue score decrease from beginning to middle season, coinciding with the team’s best performance during the season (50% of wins). However, decreased vigor score at the end compared to the beginning and middle of the season, corresponded with poor performance (33.3% wins; Silva et al., 2008). Similarly, Miranda et al. (2013) reported that the overall POMS score increased significantly in male youth soccer players after 10 weeks of training indicating greater TMD, and that the mood state disturbance was correlated with the team ranking in the championship. Mood state also appears to be related to match outcomes. Casanova et al. (2016) indicated that positive mood state (vigor) score increased after lost matches and anger score decreased after won matches during an official female association football tournament while “Iceberg” profiles were observed during all 5 matches regardless of outcome. Lowther and Lane (2002) indicated that vigor was associated with successful match performance and depression score were associated with poor match performance in a soccer team. Oliveira et al. (2009) examined the influence of match results on mood changes in professional female soccer players. They found more positive mood states among winners and more negative mood states among losers at the end of the matches, suggesting that overall mood state is related to match performance in female soccer players. Moreover, Dejongh et al. (2006) examined the relationship between in-season team performance and mood state in professional female soccer players. They showed that tension, depression, anger, and confusion scores were lower and vigor was higher when the team won indicating that poor performance was related to negative mood state in female players. In addition, Filaire et al. (2001) showed lower vigor scores when the performance of a professional male soccer team was below 50.0% wins. It is likely that competition success can lead to improved psychological states including more satisfaction, pleasure, and reduced anxiety, while the effects of defeat can produce poor well-being (Casanova et al., 2016). Alternatively, more negative mood state may decrease competitive performance.

A summary of 6 studies regarding the relationship between competitive performance and mood state variation is presented in Table 4.

**Table 4:** Findings from studies examining competitive performance and mood state.

Study	Participant (number, sex, level, age)	Condition/ duration	Aim	Results	Findings
Casanova et al. (2016)	20, female, elite (22.9 ± 4.2 years)	Competitive period (5 matches)	To compare the mood state during an official female association football tournament (5 matches)	“Iceberg” profiles of POMS were observed during all the moments of evaluation (all matches), significant decrease in vigor score after loss matches and a significant decrease in anger score after win matches	Mood state variation is influenced by match performances
Dejongh et al. (2006)	32, female, professional (19.9 ± 2.0 years)	Competitive season	To quantify the mood state changes based on match results in female soccer athletes over seasonal play	Tension, depression, anger, vigor, fatigue, confusion increased concomitantly with match losses throughout seasonal play	Negative changes in mood states. May be due to the effect of defeats during the game and the strength of opponents
Filaire et al. (2001)	17, male, professional (23.1 ± 2.2 years)	In-season period	To investigate the relationship between mood state and competitive performance	Iceberg profiles of POMS were observed during periods, which coincided with successful performance. Decrease in vigor and an increase in tension and depression were observed during periods, which coincided with decreased performance	Change in mood is related to competitive performance
Lowther and Lane (2002)	32, male, collegiate (21.2 ± 2.1 years)	Competitive period	To examine relationships between mood state and performance in a soccer team on a match-by-match basis	Relationships showed that vigor were associated with successful performance. Depression score were associated with a poor performance	Mood state was related to performance
Miranda et al. (2013)	13, male, youth players (17.0 ± 0.7 years)	Competitive period	To determine the relationship of mood state profiles and competitive performance	Relationships showed that Positive mood state were associated with outcomes	Mood state was affected by win or loss outcomes
Oliveira et al. (2009)	33, female, professional (24.2 ± 4.7 years)	Competitive period	To investigate the change of mood state at winners and in losers at the end of the match	More positive mood state found in winners and more negative mood state found in losers at the end of the match	These results suggest that mood state responds to the contest challenges among females

### 3.4. The effect of training on mood regulation

Mood regulation strategies may benefit athletic performance (Hashim et al., 2011). In fact, strong athletic performance among soccer players is associated with high positive mood scores and low negative mood scores (Hashim et al., 2011; Bijukumar, 2021). Indeed, researchers have revealed several mood regulation strategies for soccer players (Selmi et al., 2017; Bijukumar, 2021). For example, Hashim et al. (2011) compared the effects of two different relaxation techniques, known as progressive muscle relaxation and autogenic relaxation on the mood of young soccer players and showed that these relaxation techniques induced equivalent beneficial mood responses and can be used to regulate mood state (Hashim et al., 2011). Akimoto et al. (2003) studied the effect of acupuncture treatment on the mood state in 12 women soccer players during the competition period. The results indicate that acupuncture treatment has a positive effect on mood regulation with improved positive mood and reduced negative mood scores. Other less obvious mood regulation strategies may also be effective, such as choices in training programming. For example, Progressive Muscle Relaxation training (PMR)decreased mood disturbance, anger, tension, and fatigue scores in university students (Bijukumar, 2021) suggesting positive impacts on mood state. This may be a worthwhile strategy to examine among soccer players. Selmi et al. (2017) showed that SSG, a training exercise that soccer players found more enjoyable and motivating, provided better mood state regulation compared to HIIT, a less preferred training strategy, despite similar training intensities. Likewise, previous studies have shown that using motivational training exercises can reduce negative emotions and improve mood in soccer players (Hashim et al., 2011; Aydi et al., 2022). Therefore, implementing relaxation training and selection of preferred training exercises could help to regulate the mood state of athletes (Hashim et al., 2011; Bijukumar, 2021).

A summary of 4 studies examining the relationship between training and mood regulation is presented in Table 5.

**Table 5:** Findings from studies examining the relationship between training and mood regulation.

Study	Participant (number, sex, level, age)	Condition/ duration	Aim	Results	Findings
Akimoto et al. (2003)	21, female, elite (17.7 ± 1.4 years)	Competitive period	To examine the effect of acupuncture treatment on the mood state	POMS scores indicated a higher mental fatigue in the control group than in the acupuncture group	The acupuncture group suggests that acupuncture treatment had good effects on mood states during the competition period
Bijukumar (2021)	16, male, adolescent university level (15 to 18 years)	12 Sessions	To examine the effects of program of Progressive Muscle Relaxation Training (PMR) in the profile of mood states	PMR has made decreases in mood disturbance, anger, tension, and fatigue scores	PMR produce positive mood responses. This technique may be used to regulate players’ mood states
Hashim et al. (2011)	16, male, youth (14.1 ± 1.3 years)	In-season	To examine the effects of relaxation training in regulate mood states	Relaxation training results in reductions in confusion, depression, fatigue, and tension scores	Relaxation training may be used to regulate mood states
Selmi et al. (2017)	16, male, professional (24.1 ± 0.9)	Competitive period	To examine the effect training exercise with ball on mood regulation compared to HIIT	No change in POMS scores during training exercise with ball. HIIT resulted in: an increased total mood disturbance fatigue, tension decreased positive mood (vigor)	SSG ensured mood balance while HIIT produced a mood disturbance

### 3.5. Time of day and mood state

Mood state in soccer players is related to time of day and to training time (e.g., morning versus evening training) (Chtourou et al., 2012, 2014; Masmoudi et al., 2016; Irandoust et al., 2019; Hsouna et al., 2021; Masmoudi et al., 2021). In male youth soccer players, depression, RPE scores, Hooper Index, and stress were higher in the afternoon than the morning (Masmoudi et al., 2016). In some studies, training in the morning improved mood state (Masmoudi et al., 2016; Irandoust et al., 2019), whereas Masmoudi et al. (2021) showed that only depression and vigor scores, but not anger, confusion, fatigue, inter-relation, or TMD were significantly worse when training at 17:00 h compared to 08:00 h. Across these studies, exercise/training in the morning boosts physical performance and physiological status including perceived energy levels that may have positive feedback effects and perpetuate improved mood. These studies also indicated that morning activities may also confer improved mental health status and productivity throughout the day. Dietary patterns, especially periods of fasting like Ramadan, may be particularly important for time-of-day studies to consider since fasting could impact mood and performance. Chtourou et al. (2012) observed that professional soccer players felt more fatigued in the evening versus morning during Ramadan. However, among youth soccer athletes, aerobic training during Ramadan had decreased anger, confusion, depression, fatigue, tension, and TMD, and increased vigor scores regardless of whether they trained in the morning or evening (Chtourou et al., 2014).

A summary of 4 studies examining the relationship between time of day and mood in soccer players is presented in Table 6.

**Table 6:** Findings from studies examining time of day and mood regulation.

Study	Participant (number, sex, level, age)	Condition/ duration	Aim	Results	Findings
Chtourou et al. (2012)	10, male, junior (17 ± 0.5)	During Ramadan	To investigate the effect of timeofdayon mood states	Fatigue score significantly higher in the evening versus morning. Anger, confusion, and TMD scores were not significantly affected by the timeofday.	Fatigue recorded by the POMS questionnaire are higher in the afternoon during Ramadan
Chtourou et al. (2014)	30, male, young players(17.8 ± 0.5)	During Ramadan	To examine the effect of time-of-day on mood states	All subscales of mood such as anger, confusion, depression, fatigue, tension, vigor and TMD had a significant improvement following morning and evening aerobic training programs	Exercise at different times of the morning or evening can improve the mood state
Masmoudi et al. (2016)	10, male, children soccer players (14.6 ± 0.8 years)	In-season	To examine the effect of time-of-day on mood state	Negative mood (i.e., depression) significantly higher at 17:00 vs. 08:00 h	Depression recorded by the POMS questionnaire are higher in the afternoon
Masmoudi et al. (2021)	32, male, children soccer players (11 ± 0.7 years)	In-season	To investigate the effect of timeofday on mood state	Depression and vigor scores were significantly higher in the evening compared to the morning. However, anger, confusion, fatigue, inter-relation, and the TMD scores were not significantly affected by the time-of-day.	Positive mood was observed in the morning compared to the afternoon

## 4. Limitations and recommendations for further research

This work has assessed and summarized the current literature on mood state variation in soccer players with a specific focus on training loads, training modalities, and competitive performance. However, the study was not without limitations. This review only examined studies carried out in the context of soccer and not other sports disciplines. A comparison of the findings of the present study with those of other sports disciplines would be a valuable addition to the literature. Psychological responses during training for individual versus team sports would be particularly interesting. This study did not investigate the effects of Ramadan fasting on mood state since the training during Ramadan is very different from the other months of the year. Such fasting leads to physical, physiological, metabolic, and psychological changes. Moreover, this study did not examine effects of the environment (e.g., climate, altitude) on mood state. Environmental changes can also influence the physiological and psychological aspects of sport. Finally, we did not include relationships between POMS scores and other psychological states such as physical enjoyment or motivation. Such measures would be a valuable addition for mood assessment in soccer players. Future investigations examining mood state should be conducted with other sports disciplines (individual and team sports), include other mental health dimensions, and examine the influence of environmental condition to extend the findings of the present work.

## 5. Conclusion

This review examined relationships between training load/period, training exercises, and competitive performance on mood state in soccer players. It offers practical support for the use of a simple POMS measurement as part of an overall program to monitor the players’ psychological states. Results also highlighted how those training program (e.g., training load, training modality) and competitive performance are related to mood states (i.e., tension, anger, confusion, depression, fatigue, and vigor).

## 6. Practical applications

This review was conducted to summarize the current literature on the relationships between mood state in soccer players and: (1) TL and training period; (2) training exercises (e.g., modality); and (3) competitive performance. Methods of mood regulation also emerged and were examined. POMS is a simple, non-invasive, non-fatiguing, sensitive and effective measurement tool which can help coaches monitor the psychological state of soccer players. Mood state assessment can be useful in predicting mental fatigue and quantifying emotional state of players during training. It follows that the technical staff of soccer teams should bear in mind that an increase in TL (i.e., intensity and volume of training), fatigue accumulation and poor recovery negatively affect the mood state of players.

Monitoring mood state variation during training may also help predict player performance. This strategy could be useful for coaches to determine when to implement techniques to improve stress coping and to avoid potential negative impacts of extended periods of high TL such as anxiety and loss of concentration. Better identification of athletes’ psychological states allows the technical staff to select programming and strategies to achieve better outcomes during training and competition.

## Author contributions

All authors listed have made a substantial and intellectual contribution to the work, and approved it for publication.

## Conflict of interest

The authors declare that the research was conducted in the absence of any commercial or financial relationships that could be construed as a potential conflict of interest.

## Publisher’s note

All claims expressed in this article are solely those of the authors and do not necessarily represent those of their affiliated organizations, or those of the publisher, the editors and the reviewers. Any product that may be evaluated in this article, or claim that may be made by its manufacturer, is not guaranteed or endorsed by the publisher.
